# Zinc Alpha 2 Glycoprotein as an Early Biomarker of Diabetic Nephropathy in Type 2 Diabetes Mellitus Patients

**DOI:** 10.7759/cureus.36011

**Published:** 2023-03-11

**Authors:** Satyendra K Sonkar, Akash Gupta, Gyanendra K Sonkar, Kauser Usman, Vivek Bhosale, Satish Kumar, Sharad Sharma

**Affiliations:** 1 Medicine, King George's Medical University, Lucknow, IND; 2 Biochemistry, King George's Medical University, Lucknow, IND; 3 Clinical and Experimental Medicine, Central Drug Research Institute (CDRI), Lucknow, IND; 4 Toxicology, Central Drug Research Institute (CDRI), Lucknow, IND

**Keywords:** glycated hemoglobin (hba1c), adipokine, anti-inflammatory, egfr, microalbuminuria

## Abstract

Background and objectives

Microalbuminuria is an early sign of diabetic nephropathy (DN). However, pathological abnormalities occur before the onset of microalbuminuria. Renal impairment progresses in about 50% of cases in type 2 diabetes mellitus (T2DM) without significant albuminuria. Diabetes mellitus (DM) is linked with obesity, metabolic syndrome, and lifestyle changes, where adipokines play an important role. Zinc alpha 2 glycoprotein (ZAGP) is an adipokine, and in this study, it was assessed as a potential biomarker for early DN as well as its progression.

Materials and methods

This study was a cross-sectional case-control study conducted at a tertiary hospital in northern India. T2DM patients aged 18-65 years old were included in the study and were divided into four groups based on their albuminuria level. This study included 160 participants, with 40 participants in each group. Group I included healthy volunteers, while Groups II, III, and IV were normoalbuminuric, microalbuminuric, and macroalbuminuric diabetic patients, respectively.

The groups were evaluated for demographic variables, biochemical parameters, urine albumin-creatinine ratio (UACR), and serum ZAGP. Data between the groups were compared statistically.

Results

This study included 160 participants, with 40 participants in each group. There was a significant difference between the groups based on the serum ZAGP (p<0.001). Serum ZAGP was significantly negatively correlated with serum creatinine, glycosylated hemoglobin (HbA1c), serum cholesterol, serum triglyceride, low-density lipoprotein (LDL) cholesterol, and UACR. ZAGP was positively correlated with the estimated glomerular filtration rate (eGFR).

Conclusion

The present study showed that ZAGP was an early biomarker of diabetic nephropathy, and its value decreased as DN progressed. It also suggested that ZAGP, an adipokine, has an anti-inflammatory mechanism of action and its depletion worsens the disease.

## Introduction

The number of diabetes mellitus (DM) patients is increasing globally because of the heightened rates of obesity, metabolic syndrome, sedentary lifestyle, unhealthy dietary pattern, lack of exercise, and consumption of tobacco and alcohol. About 95% of DM is due to type 2 diabetes mellitus (T2DM). Diabetic kidney disease (DKD) is responsible for the bulk of diabetes-related morbidity and mortality [1]. In India, the prevalence of DKD is more common and is around 34.4% [2]. DM is responsible for about 50% of the cases of end-stage renal disease (ESRD) and of which the maximum number of patients are of T2DM. Early diagnosis and management of diabetic nephropathy (DN) could therefore slow the progression of DKD and prevent the premature cardiovascular mortality associated with it [3].

The most common method of detecting the early signs of DN is the presence of microalbuminuria, where predominantly glomerulus is involved [4]. However, pathological abnormalities have been reported to occur before the onset of microalbuminuria. Apart from this classical DN, there is another non-dominant glomerular disease leading to renal dysfunction. Normoalbuminuric chronic kidney disease (NA-CKD) has become a prevalent variant of renal impairment in diabetes, accounting for 50% of cases of DKD [5]. This is characterized by a decline in the glomerular filtration rate in the absence of a preceding or accompanying elevation of albuminuria. 

T2DM is linked with visceral adiposity. Adipose tissue, which acts as an endocrine organ, synthesizes and secretes different adipokines that play an important role as pro- or anti-inflammatory mediators. In T2DM the accumulation of fat in the adipose tissue leads to increased lipotoxicity which causes both beta-cell dysfunction and insulin resistance [6]. Zinc Alpha 2 glycoprotein (ZAGP), an adipokine that acts as a lipid-mobilizing factor, plays a crucial role in lipolysis in adipose tissue [7]. ZAGP is postulated to inhibit lipogenic enzymes and enhance the action of lipolytic enzymes in adipose tissue through various pathways. A reduction in the levels of ZAGP could lead to lower rates of lipolysis and subsequent lipotoxicity, causing endothelial dysfunction and microvascular complications [8]. ZAGP has also been considered a marker of tubular damage in the kidney [9,10].

In this study, we assessed ZAGP as an early biomarker for DN compared to microalbuminuria and its role in the progression of DKD. Previous studies on ZAGP have mainly focused on its role in tubular damage; however, the present study considers its role as an anti-inflammatory adipokine.

## Materials and methods

This study was a cross-sectional, case-control study conducted at a tertiary hospital in northern India. The study population was divided into four groups based on the participants’ level of albuminuria.

First group: The control group (Group I) were normal healthy volunteers.

Second group: Normoalbuminuric diabetic group (Group II) (urine albumin creatinine ratio <30 mg/gm creatinine)

Third group: DN group with microalbuminuria (Group III, urine albumin creatinine ratio from 30 to 300 mg/gm creatinine)

Fourth group: DN group with macroalbuminuria (Group IV, urine albumin creatinine ratio >300 mg/gm creatinine)

A sample size of 40 participants in each group was enrolled using the software G Power analysis (Heinrich-Heine-Universität Düsseldorf, Düsseldorf, Germany; http://www.gpower.hhu.de/) with a medium effect size of 0.30, 95% confidence interval, and 80% power of the study.

T2DM patients, per the American Diabetes Association (ADA) criteria of ages 18-65 years and who gave written informed consent, were included in the study. Staging of DKD was done based on the kidney disease: Improving Global Outcomes (KDIGO) guidelines. Patients with acute on chronic kidney diseases, polycystic kidney, hepatic diseases, malignancy, inflammatory conditions, and sepsis were excluded.

The study was approved by the Institutional Ethics Committee. The procedure was carried out based on the Declaration of Helsinki and the International Council for Harmonization-Good Clinical Practice (ICH-GCP).

The clinical and laboratory findings were compared between the four groups. Demographic and clinical data (e.g., comorbidities, duration of type 2 diabetes, and blood pressure) were recorded. Blood samples were collected for testing complete blood count, including sodium (Na+), potassium (K+), urea/creatinine (Cr), glycosylated hemoglobin (HbA1c), serum cholesterol (CHL), serum triglyceride (TG), serum low-density lipoprotein cholesterol (LDL), serum very low-density lipoprotein (VLDL), serum high-density lipoprotein (HDL), serum phosphate (PO4), serum uric acid (UA), and serum ZAGP. A urine examination was done for the albumin-creatinine ratio (UACR). UACR was reported as mg albumin/g creatinine. Commercially available enzyme-linked immunosorbent assay (ELISA) kits were used to measure ZAGP. The plasma and serum were centrifuged and frozen at -70 °C until further laboratory analysis. The estimated glomerular filtration rate (eGFR) was calculated using the Modification of Diet in Renal Disease formula (MDRD):

eGFR = 175 × (serum creatinine)^-1.154^ × (age)^-0.203^ × (0.742 only if female) × (1.212 only if black)

Statistical analysis

Baseline characteristics were assessed using standard descriptive statistics. The normality of data was tested by the Kolmogorov-Smirnov test. If the data were not found to be continuous, then the non-parametric test was used. Continuous variables were presented as mean ± standard deviation. Categorical variables were presented in number and percentage (%). Quantitative variables were compared using an F-test between groups, while qualitative variables were compared using the Chi-Square test/Fisher’s exact test where they are appropriate. Here, a p-value of < 0.05 was considered statistically significant. The one-way analysis of variance (ANOVA) was also used to determine whether there were statistically significant differences between the means of two or more independent (unrelated) groups. The Pearson correlation coefficient was applied to measure the strength of a linear association between two variables, where the value r = 1 means a perfect positive correlation, and the value r = -1 means a perfect negative correlation. The data were entered into a Microsoft Excel spreadsheet, and the analysis was done using Statistical Product and Service Solutions (SPSS) (IBM SPSS Statistics for Windows, Version 29.0, Armonk, NY).

## Results

In the present study, 160 subjects (47.5% men and 52.5% women) were registered. The mean age was 47.2 + 9.1 years. Among the subjects, 120 were diabetic patients, and 40 were healthy controls. The majority of the patients (48 %) were in stage G3 of DKD, while 9.2%, 15%, 20%, and 7.5% were in stages G1, G2, G4, and G5, respectively. The mean duration of T2DM among the cases was 6.93 + 5.4 years. Among the diabetic patients, 77 (64.2%) had hypertension, 19 (11.8%) had coronary artery disease, and seven (4.4%) had a cerebrovascular accident. A baseline comparison between the diabetic groups based on the clinical and biochemical parameters was made. The maximum duration of T2DM was noted in the diabetic macroalbuminuria group (8.05 + 4.5 years). Most numbers of hypertensive patients were also noted in this group (80%). A significant difference between the groups in hemoglobin level (HB), total leucocyte count (TLC), platelet count (PLT), HbA1c, eGFR, urea, Cr, CHL, TG, LDL cholesterol, VLDL cholesterol, PO4, and UA was found (Table 1). The ZAGP was at a maximum level in the healthy control group (mean = 148.17 + 25.5 ng/mL) and minimum in the diabetic macroalbuminuria group (mean = 86.3 + 21.4 ng/mL) (p < 0.001) (Table 1).

**Table 1:** Baseline comparison of groups based on biochemical parameters hemoglobin (HB); total leucocyte count (TLC); platelet count (PLT); creatinine (CR); glycosylated hemoglobin (HbA1c); albumin creatinine ratio (ACR); cholesterol (CHL); triglyceride (TG); low-density lipoprotein cholesterol (LDL); high-density lipoprotein (HDL); very low-density lipoprotein (VLDL); phosphate (PO4); zinc alpha 2 glycoprotein (ZAGP); analysis of variance (ANOVA); estimated glomerular filtration rate (eGFR)

Variables	Normal non-diabetic (N=40)		Diabetic microalbuminuria (N=40)		Diabetic normoalbuminuria (N=40)		Diabetic macroalbuminuria (N=40)		ANOVA	
	Mean	SD	Mean	SD	Mean	SD	Mean	SD	F value	p-value
HB (gm/dL)	11.30	1.37	10.73	1.81	11.35	1.29	9.60	1.19	12.78	<0.001
TLC (per mm^3^)	8136	3138.25	7821	2752.41	8339	2177.29	10480	3472.12	6.84	<0.001
PLT (lakhs/mm^3^)	2.08	0.74	1.84	0.79	1.88	0.76	2.31	0.93	2.89	<0.05
UREA (mg/dL)	39.0	16.2	67.9	79.1	34.2	14.1	52.2	26.5	4.92	<0.05
CR (mg/dL)	1.14	0.35	1.70	0.53	1.10	0.42	2.56	1.29	32.93	<0.001
HbA1c (percentage)	5.19	0.48	7.92	1.04	7.11	1.84	8.79	1.73	48.63	<0.001
URINE ACR (mg/gm of creatinine)	10.55	10.07	214.30	180.59	14.88	8.37	925.62	884.89	36.84	<0.001
eGFR (ml/min/1.73m^2^)	77.22	66.68	43.62	19.30	81.62	85.72	29.95	19.26	8.17	<0.001
CHL (mg/dL)	164.7	32.4	176.4	48.1	161.3	38.6	209.5	41.1	11.79	<0.001
TG (mg/dL)	159.5	55.6	182.6	73.5	154.3	56.8	219.0	78.0	7.786	<0.001
LDL (mg/dL)	86.5	27.0	86.5	42.0	83.3	34.5	114.1	41.4	6.16	<0.001
HDL (mg/dL)	41.2	13.4	43.0	17.2	45.7	17.0	51.0	27.6	1.91	0.13
VLDL (mg/dL)	31.7	11.7	37.9	15.0	30.7	11.4	44.8	14.9	9.41	<0.001
PO4 (mg/dL)	N/A	N/A	6.18	0.95	5.64	1.11	7.38	1.02	29.61	<0.001
URIC ACID (mg/dL)	N/A	N/A	6.2	0.8	5.9	0.9	7.1	1.3	14.62	<0.001
ZAGP (ng/mL)	148.2	25.50	126.8	13.46	111.3	14.07	86.3	21.36	22.54	<0.001

It was found that ZAGP is significantly negatively correlated with age, duration of T2DM, systolic blood pressure (SBP), HB, Cr, HbA1c, CHL, TG, LDL cholesterol, UACR, and PO4, while it was positively correlated with eGFR as shown in Table 2.

**Table 2:** Linear correlation between zinc alpha 2 glycoprotein and other variables glycosylated hemoglobin (HbA1c); blood pressure (BP); estimated glomerular filtration rate (eGFR); diabetes mellitus (DM); zinc alpha 2 glycoprotein (ZAGP); creatinine (CR); albumin creatinine ratio (ACR); phosphate (PO4); hemoglobin (HB); total leucocyte count (TLC); platelet count (PLT); cholesterol (CHL); triglyceride (TG); low-density lipoprotein cholesterol (LDL); high-density lipoprotein (HDL); very low-density lipoprotein (VLDL)

ZAGP	Age	Duration of DM	Systolic BP	CR	HbA1c	eGFR	Urine ACR	P04
Pearson Correlation (r)	-0.18	-0.34	-0.26	-0.41	-0.33	0.52	-0.44	-0.62
p-value	<0.05	<0.001	<0.001	<0.05	<0.05	<0.001	<0.001	<0.001
	HB	TLC	PLT	CHL	TG	LDL	HDL	VLDL
Pearson Correlation (r)	0.303	-0.254	-0.065	-0.309	-0.279	-0.256	-0.147	-0.319
p-value	< .001>	<0.001	0.411	<0.001	<0.001	<0.001	0.064	<0.001

However, on multiple regression, ZAGP was significantly associated with Cr, PO4, HbA1c, CHL, and LDL (Table 3).

**Table 3:** Multiple linear regression analysis with ZAGP as a dependent variable hemoglobin (HB); total leucocyte count (TLC); platelet count (PLT); creatinine (CR); glycosylated hemoglobin (HbA1c); albumin creatinine ratio (ACR); cholesterol (CHL); triglyceride (TG); low-density lipoprotein cholesterol (LDL); high-density lipoprotein (HDL); very low-density lipoprotein (VLDL); phosphate (PO4); zinc alpha 2 glycoprotein (ZAGP)

Model	Unstandardized coefficients		Standardized coefficients	t	Sig.
	B	Std. Error	Beta		
(Constant)	204.524	20.657		9.901	<0.001
HB	-0.508	1.108	-0.035	-0.458	0.648
TLC	-0.001	0.001	-0.071	-1.014	0.313
PLT	-0.670	1.875	-0.024	-0.357	0.722
UREA	0.008	0.030	0.017	0.261	0.795
CR	-7.468	2.042	-0.327	-3.656	<0.001
HbA1c	-3.213	0.961	-0.234	-3.344	0.001
URINE ACR	-0.003	0.003	-0.085	-1.135	0.259
CHL	0.203	0.095	0.406	2.140	0.035
TG	-0.074	0.049	-0.233	-1.507	0.135
LDL	-0.206	0.086	-0.364	-2.388	0.019
HDL	-0.145	0.098	-0.131	-1.470	0.145
VLDL	-0.017	0.247	-0.011	-0.071	0.944
PO4	-6.658	1.393	-0.356	-4.779	<0.001
URIC ACID	0.152	1.449	0.008	0.105	0.916
Dependent Variable: ZAGP					

## Discussion

Adipokines play an important role in the pathogenesis of DM which is related to adiposity. Adipose tissue has been identified as an endocrine organ secreting adipokines involved in metabolic and inflammatory pathways [11]. ZAGP is a novel adipokine; its expression in adipose tissue positively correlates with adiponectin expression [12,13].

ZAGP has been suggested to be an adipokine that acts as a lipid-mobilizing factor playing a role in lipolysis [7]. Decreasing ZAGP levels are associated with the onset of lipotoxicity, which is supported by previous studies showing its positive association with adiponectin and negative association with tumor necrosis factor-alpha, suggesting a possible anti-inflammatory role of ZAGP [12,13]. This role was also found in a study in which ZAGP was reduced in early sepsis, and it increased with clinical recovery [14].

It was also found that ZAGP was negatively correlated with Cr, HbA1c, and UACR and positively correlated with eGFR. Increased levels of Cr, HbA1c, UACR, and decreased eGFR suggest deterioration of the disease. In addition, the study showed a significant difference between the four groups based on biomarker ZAGP, with values decreasing as DN progressed.

Thus, ZAGP acts as a negative biomarker and decreases as the disease progresses. This contradicts earlier studies on ZAGP, where it was found that ZAGP levels increased in DN patients and had a positive correlation with UACR and a negative correlation with eGFR [9,15-18].

In this study, T2DM patients had greater blood ZAGP levels than the control subjects. High HbA1c patients also had a significantly low ZAGP level, which can be attributed to the reduction in the ZAGP levels and could lead to lower rates of lipolysis and subsequent accumulation of lipids. This resulting lipotoxicity is recognized as resulting in cellular dysfunction, including impaired glucose uptake, pancreatic beta-cell dysfunction, and increasing the severity of diabetes [19].

Hypertension was present in 50% of the diabetic normoalbuminuric group, 62.5% of the diabetic microalbuminuric group, and 80% of the diabetic macroalbuminuric group, where there was a significant negative correlation between SBP and ZAGP. The explanation for this is that ZAGP is a novel adipokine that is reduced in patients with hypertension [20], decompensated heart failure, and dyslipidemia [21]. Diabetes concomitant with hypertension markedly increases the risk of cardiovascular mortality associated with endothelial dysfunction and inflammation. Therefore, ZAGP is an anti-inflammatory adipokine; its values decrease with increased inflammation. In a previous study, ZAGP levels were significantly lowered in patients with hypertension and negatively correlated with obesity compared to the general population [18]. Hypertension is approximately twice as prevalent in patients with diabetes compared to the general population [22].

Moreover, there was a significant difference between the four groups (p < 0.05) based on lipid profile, such that there was a significant increase in CHL and LDL in the macroalbuminuric group, suggestive of dyslipidemia. There was a significant depletion of ZAGP compared to normal healthy controls and the diabetic normoalbuminuric group. A systematic review and meta-analysis investigating the associations between ZAGP and dyslipidemia have revealed that circulating ZAGP was lower in individuals with dyslipidemia compared to metabolically healthy controls [12].

Drugs, such as Canagliflozin [23] and Sitagliptin [16], can modulate the value of ZAGP, which suggests an improved outcome with an increase in ZAGP level. Drugs acting through Sodium-glucose co-transporter 2 (SGLT2) inhibitors would increase the expression of ZAGP leading to the downregulation of chronic inflammation and the reduction of oxidative stress in obese subjects [23]. Similarly, drugs acting through the Glucagon-like peptide-1 (GLP-1) pathway would prolong the action of GLP-1, leading to an improvement in glucose metabolism and insulin sensitivity [16]. The value of ZAGP also significantly decreased in the normoalbuminuric diabetic group compared to healthy controls, implying that it is a useful early biomarker for non-albuminuric DKD.

## Conclusions

The present study shows that ZAGP could be an early novel biomarker of DKD. Lower levels of ZAGP were associated with the deterioration of renal function and disease progression; hence, it plays an essential role as an anti-inflammatory adipokine. More extensive prospective studies are required to ascertain the role of ZAGP and its therapeutic potential.

## References

[1] Thomas B (2019). The global burden of diabetic kidney disease: time trends and gender gaps. Curr Diab Rep.

[2] Hussain S, Jamali MC, Habib A, Hussain MS, Akhtar M, Najmi AK (2021). Diabetic kidney disease: an overview of prevalence, risk factors, and biomarkers. Clin Epidemiology Glob Health.

[3] Thomas MC, Brownlee M, Susztak K (2015). Diabetic kidney disease. Nat Rev Dis Primers.

[4] Persson F, Rossing P (2018). Diagnosis of diabetic kidney disease: state of the art and future perspective. Kidney Int Suppl (2011).

[5] Klimontov VV, Korbut AI (2019). Albuminuric and non-albuminuric patterns of chronic kidney disease in type 2 diabetes. Diabetes Metab Syndr.

[6] Cusi K (2010). The role of adipose tissue and lipotoxicity in the pathogenesis of type 2 diabetes. Curr Diab Rep.

[7] Bing C, Trayhurn P (2009). New insights into adipose tissue atrophy in cancer cachexia. Proc Nutr Soc.

[8] Hassan MI, Waheed A, Yadav S, Singh TP, Ahmad F (2008). Zinc alpha 2-glycoprotein: a multidisciplinary protein. Mol Cancer Res.

[9] Wang Y, Li YM, Zhang S, Zhao JY, Liu CY (2016). Adipokine zinc-alpha-2-glycoprotein as a novel urinary biomarker presents earlier than microalbuminuria in diabetic nephropathy. J Int Med Res.

[10] Kamel MF, Nassar M, Elbendary A (2022). The potential use of urinary transferrin, urinary adiponectin, urinary Retinol Binding Protein, and serum zinc alpha 2 glycoprotein levels as novel biomarkers for early diagnosis of diabetic nephropathy: a case-control study. Diabetes Metab Syndr.

[11] Ouchi N, Parker JL, Lugus JJ, Walsh K (2011). Adipokines in inflammation and metabolic disease. Nat Rev Immunol.

[12] Pearsey HM, Henson J, Sargeant JA (2020). Zinc-alpha2-glycoprotein, dysglycaemia and insulin resistance: a systematic review and meta-analysis. Rev Endocr Metab Disord.

[13] Bao Y, Bing C, Hunter L, Jenkins JR, Wabitsch M, Trayhurn P (2005). Zinc-alpha2-glycoprotein, a lipid mobilizing factor, is expressed and secreted by human (SGBS) adipocytes. FEBS Lett.

[14] Welters ID, Bing C, Ding C, Leuwer M, Hall AM (2014). Circulating anti-inflammatory adipokines High Molecular Weight Adiponectin and Zinc-α2-glycoprotein (ZAG) are inhibited in early sepsis, but increase with clinical recovery: a pilot study. BMC Anesthesiol.

[15] Sörensen-Zender I, Beneke J, Schmidt BM, Menne J, Haller H, Schmitt R (2013). Zinc-alpha2-glycoprotein in patients with acute and chronic kidney disease. BMC Nephrol.

[16] Tian M, Liang Z, Liu R (2016). Effects of sitagliptin on circulating zinc-α2-glycoprotein levels in newly diagnosed type 2 diabetes patients: a randomized trial. Eur J Endocrinol.

[17] Xu L, Yu W, Niu M (2017). Serum ZAG levels were associated with eGFR mild decrease in T2DM patients with diabetic nephropathy. Int J Endocrinol.

[18] Elsheikh M, Elhefnawy KA, Emad G, Ismail M, Borai M (2019). Zinc alpha 2 glycoprotein as an early biomarker of diabetic nephropathy in patients with type 2 diabetes mellitus [Article in Portuguese]. J Bras Nefrol.

[19] Yang H, Li X (20121). The role of fatty acid metabolism and lipotoxicity in pancreatic β-cell injury: identification of potential therapeutic targets. Acta Pharmaceutica Sinica B.

[20] Zhu HJ, Wang XQ, Pan H (2014). Serum levels of the adipokine zinc-α2-glycoprotein are decreased in patients with hypertension. ISRN Endocrinol.

[21] Yeung DC, Lam KS, Wang Y, Tso AW, Xu A (2009). Serum zinc-alpha2-glycoprotein correlates with adiposity, triglycerides, and the key components of the metabolic syndrome in Chinese subjects. J Clin Endocrinol Metab.

[22] Epstein M, Sowers JR (1992). Diabetes mellitus and hypertension. Hypertension.

[23] Kabil SL, Mahmoud NM (2018). Canagliflozin protects against non-alcoholic steatohepatitis in type-2 diabetic rats through zinc alpha-2 glycoprotein up-regulation. Eur J Pharmacol.

